# Development of a novel hybrid securing actuator for a self-securing soft robotic hand exoskeleton

**DOI:** 10.3389/frobt.2023.1164819

**Published:** 2023-07-25

**Authors:** Luis Hernandez-Barraza, Azmall Fraiszudeen, Daniel Lim Yuan Lee, Raye Chen-Hua Yeow

**Affiliations:** ^1^ Evolution Innovation Lab, Advanced Robotics Centre, National University of Singapore, Singapore, Singapore; ^2^ Department of Biomedical Engineering, National University of Singapore, Singapore, Singapore

**Keywords:** wearable hand exoskeleton, self-wearing glove, hybrid soft glove, rehabilitation, actuators

## Abstract

The development of soft robotic hand exoskeletons for rehabilitation has been well-reported in the literature, whereby the emphasis was placed on the development of soft actuators for flexion and extension. Little attention was focused on developing the glove interface and attachments of actuators to the hand. As these hand exoskeletons are largely developed for personnel with impaired hand function for rehabilitation, it may be tedious to aid the patients in donning and doffing the glove, given that patients usually have stiff fingers exhibiting high muscle tone. To address this issue, a hybrid securing actuator was developed and powered pneumatically to allow for rapid securing and release of a body segment. As a proof of concept, the actuator was further adapted into a self-securing glove mechanism and assembled into a complete self-securing soft robotic hand exoskeleton with the attachment of bidirectional actuators. Our validation tests show that the self-wearing soft robotic hand exoskeleton can easily conform and secure onto the human hand and assist with manipulation tasks.

## 1 Introduction

The advent of soft robotics research in recent years is a response to the increasing need for automation, efficiency, and human-robot collaboration, especially in healthcare (Rotella et al., 2009; Zhu et al., 2020; Xiong et al., 2021; Miller-Jackson et al., 2022). A major area of research is developing robotic hand exoskeletons as finger exercise therapy devices augment standard occupational therapy using hard (Yeow et al., 2014; Keong and Hua, 2018). and soft robotics (Low et al., 2015; Meng et al., 2017; Chu and Patterson, 2018; Shahid et al., 2018; Tang et al., 2021; Kim et al., 2022; Rakhtala and Ghayebi, 2022). Especially in the latter field of research, several groups have contributed to this endeavor. For instance, Polygerinos et al. developed a hydraulic grip glove that utilizes fiber-reinforced actuators mechanically programmable to generate motion paths similar to the kinematics of the human thumb and fingers. (Polygerinos et al., 2015a; Polygerinos et al., 2015b). Noritsugu et al. developed a power assist glove that utilizes sheet-like curved rubber muscles for hand-grasping applications (Noritsugu et al., 2004). Yap et al. have developed soft-elastomeric actuators and fabric-based hand exoskeletons designed to rehabilitate the hand (Yap et al., 2016a; Yap et al., 2016b; Yap et al., 2016c; Yap et al., 2017a). In these works, the hand exoskeletons function as assistive tools in aiding hand-impaired persons to perform physical practice in rehabilitation, primarily in stroke.

A prevailing feature of the developed hand exoskeletons in literature is the incorporation of actuators mounted on gloves that the user wears. The actuators are the driving force in aiding the flexion and extension of the impaired fingers, while the glove acts as an interface between the hand and the actuator, holding the actuators in place to act upon the user’s hand. In most hand exoskeleton designs, much emphasis is placed on the actuator’s functionality, whereas soft actuators are characterized in great detail concerning their bending characteristics and force output (Mosadegh et al., 2014; Polygerinos et al., 2015c; Yap et al., 2016c). On the other hand, the glove component tends to be cursorily treated and is seen simply as a means of attaching the actuators to the hand. However, a hallmark of the paretic hand in stroke includes high muscle tone and stiffness, known as spasticity (Chen et al., 2009; Yap et al., 2016b). This complicates the process of helping stroke survivors don and doff these hand exoskeletons. This is because aiding the stroke survivors to don the glove requires every finger of the patient to be carefully inserted into the corresponding finger sleeve of the glove, which can be an arduous task, as their spastic fingers may not easily fit into the finger sleeves. As such, before using the hand exoskeletons in rehabilitation, there is usually a need for an additional step of stretching the fingers of these stroke survivors, performed by a trained caregiver, to relax their fingers enough to be able to insert them within the glove.

The concept of self-securing and self-adjustment has been explored in several application domains, such as adaptive clothing for the disabled (Na, 2007), aerospace garments (Clarke et al., 2016; Holschuh and Newman, 2016), sports (Myers et al., 2019), and rehabilitation (Cai et al., 2009; Cempini et al., 2012; Brenneis et al., 2018). The idea that another device or accessory can be quickly and firmly secured to the human body simply by positioning the body segment in place and activating a locking mechanism lends itself well to the problem of helping caregivers and clinicians don or doff hand exoskeletons for stroke patients. In this paper, a novel endoskeleton-supported hybrid securing actuator is introduced, which serves as an interface to mechanically couple the device and body segment and allows for quick insertion and release of body segments from the device in transition. As a proof of concept of its practical application, it is further developed into a self-securing glove to be used with our previously (Low et al., 2017) developed soft bidirectional actuators.

## 2 Materials and methods

### 2.1 Actuator design

**FIGURE 1:**
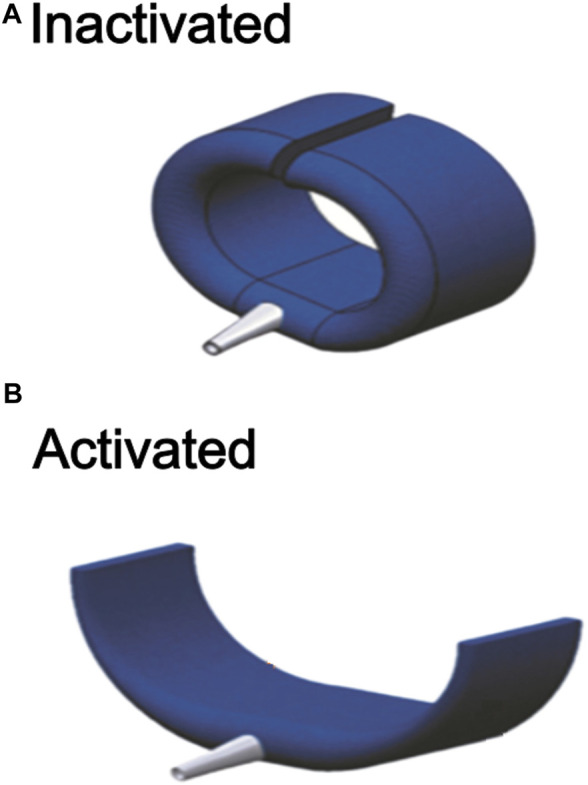
**(A)** The completed actuator in its activated uncurled state. **(B)** The completed actuator in its inactivated curled state.

The securing actuator developed in this study is a disjointed circular ring in its inactivated state, which passively encircles a body, tethering itself to the body (Figure 1A). Activation of the actuator causes the form to change to a straightened one, which releases the body it is tethered to (Figure 1B). The actuator is designated as a means for coupling a wearable accessory to the human body, where it can be easily activated and de-activated to release and secure the accessory. Specifically, it is designed to work with other soft robotic actuators and soft exoskeletons. The main use case explored in this study sees the securing actuator acting as an interface to secure soft finger actuators to the hand. This is demonstrated in the later section.

The securing actuator is constructed by encasing a flexible polyvinyl chloride (PVC) endoskeleton, shaped as a disjointed ring, within a fabric air bladder, hence its hybrid nature. The PVC endoskeleton supports the securing actuator mechanically and defines the shape taken by the actuator, either the curled or uncurled configuration. On the other hand, the fabric air bladder component is pneumatically powered and can take two forms. In its inactivated state (without air pressure within its chambers), it is flaccid and compliant. Upon activation by air pressure, it stiffens, straightens into a straight beam-like structure, and resists deformation. Switching between curled or uncurled states of the actuator involves the interplay of two opposite forces—the curled mechanical state of the supporting endoskeleton in the absence of external forces, much like a coiled spring, and the opposing straightening force provided by a pneumatically-powered air bladder upon activation. Without external forces, the actuator assumes its disjointed ring or C-shape. This is the non-activated or ‘secured’ state (Figure 1A). The fabric air bladder is activated pneumatically, stiffening and straightening. As a result, it provides an opposing force that overcomes the curled configuration of the endoskeleton ring and pulls it open (Figure 1B), causing it to uncurl. This forms the activated or ‘released’ state of the securing actuator, allowing the body segment it is securing to be inserted or removed from the securement.

### 2.2 Actuator fabrication

Fabrication of the securing actuator involves attaching the PVC endoskeleton ring atop the fabric air bladder component and sealing it with another layer of fabric. Thermoplastic Polyurethane (TPU)-coated polyester fabric (Jiaxing Inch ECO materials Co., LTD., Zhejiang, China) was used to fabricate the air bladder component to attach the exoskeleton to the air bladder. TPU melts and forms an adhesive under heat and pressure. The TPU coating is on one side of the fabric, allowing the air bladder to be sealed by placing two TPU-coated sides against each other and applying a heat press. The supplied heat and pressure melt the TPU and cause the two TPU-coated surfaces to adhere to another one, forming the seal (Hernandez Barraza et al., 2023). The air channel within the bladder is created by preventing the TPU-coated surfaces from forming a seal at the desired region. This is achieved by patterning a layer of masking tape over the TPU-coated surface.

**FIGURE 2:**
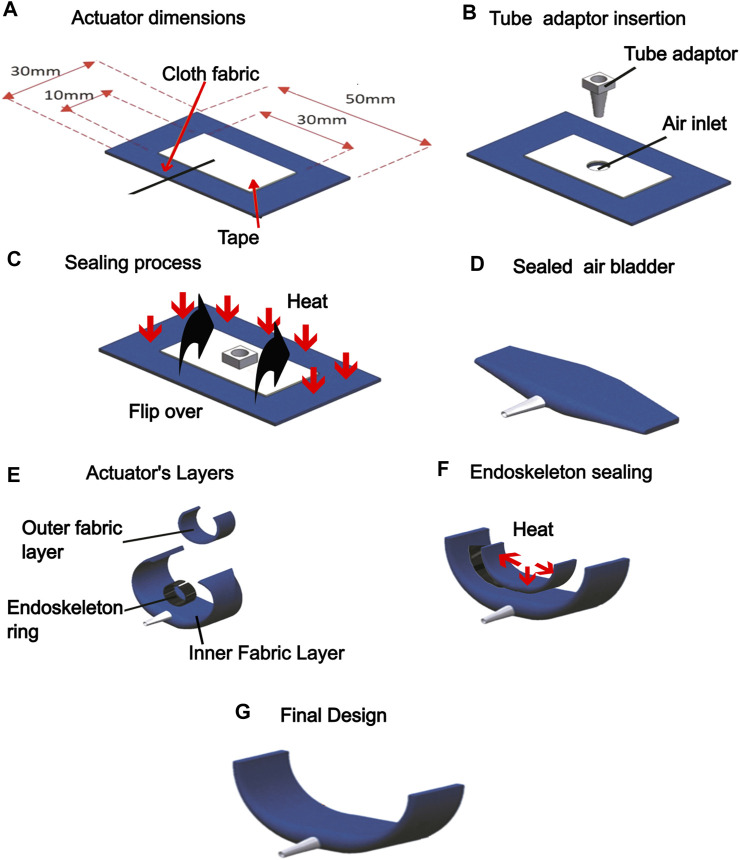
Fabrication of hybrid securing actuator. **(A)** The air channel is dimensioned within the TPU-coated polyester fabric and masked with a masking tape. **(B)** An air inlet was punctured to allow access to the air channel and the tube adaptor was inserted **(C)** An air inlet was punctured to allow access to the air channel, and the tube adaptor was inserted. **(D)** Heat is applied to the fabric after it is folded in half to seal the actuator. **(E)** The plastic endoskeleton is joined atop the bladder using hot glue adhesive. **(F)** An additional fabric layer is heat sealed to conceal the plastic endoskeleton. **(G)** The completed actuator.

The process of fabrication is outlined in Figure 1. First, the TPU-coated fabric sheet is dimensioned and cut out. Masking tape is pated on the TPU-coated surface of the polyester fabric, demarcating the air channel (Figure 2A). an air inlet is introduced in the fabric. A pneumatic tube adaptor is inserted into the air inlet, allowing pneumatic access to the air bladder (Figure 2B, C). The fabric is then folded in half breadthwise, and heat-pressing is applied, which melts TPU-coated regions and seals the air bladder (Figure 2D). With the air bladder sealed, the PVC endoskeleton is joined atop and in the middle of the bladder using a hot-melt thermoplastic adhesive (Figure 2E). A separate strip of TPU-coated polyester fabric sheet was then placed atop the endoskeleton bonded to the bladder and heat-pressed onto the air bladder, enveloping the endoskeleton between the fabric strips (Figure 2F) and the fabric bladder to give the completed actuator showed in Figure 2G.

### 2.3 Characterization tests

The securing actuator was characterized in terms of two defined quantities: The locking force and opening width. The locking force was defined as the force needed to overcome the securing forces conferred by the endoskeleton to hold the enveloped body segment in place. The opening width was the linear displacement that can be inserted and secured.

### 2.4 Locking force

**FIGURE 3:**
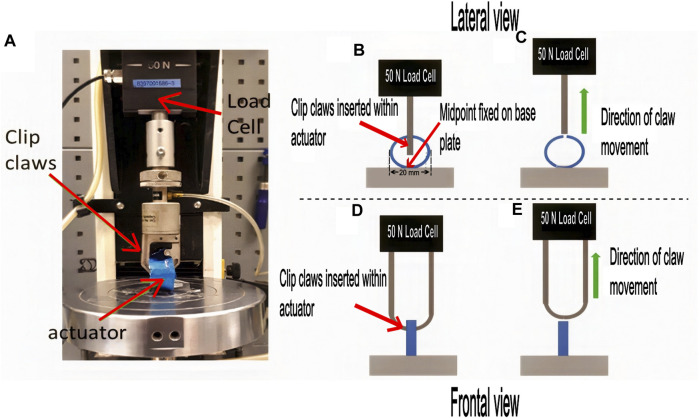
Locking force characterization experiment. **(A)** shows the Instron machine set up to measure the locking force of the inactivated securing actuator in its ‘secured’ configuration. **(B)** and **(C)** shows a schematic of the side view of the setup. **(D, E)** shows that of the front view of the setup.

The locking force was measured using an Instron machine (Instron, Measurement Specialties Inc., United States) with a mounting platform and 50 N tension load cell (Figure 3A). The steps for taking a single measurement of locking force were as follows: first, the inactivated actuator was mounted on the platform, with its midpoint fixed on the baseplate (Figure 3B, D). The claws of the load cell were inserted within the securing region of the actuator. After setting up, the trial starts by moving the claw upwards at a fixed velocity of 8 mm/min until it overcomes the locking configuration of the endoskeleton and dislodges (Figure 3C, E). During this process, the actuator’s force exerted on the claws is measured continuously to determine the force profile and the maximum force encountered, representing the locking force.

### 2.5 Opening width

**FIGURE 4:**
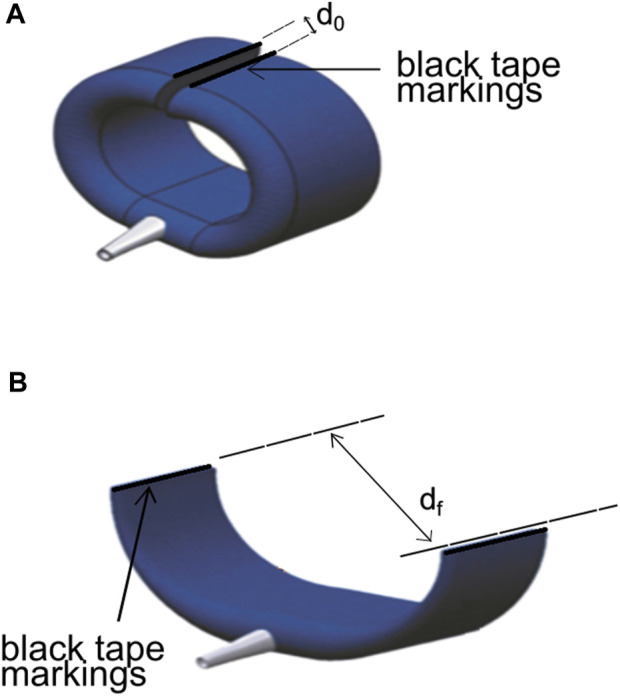
Actuator showing the black tape markings attached for the opening width test. **(A)** Actuator in the deflated state at d_0_. **(B)** Actuator showing the increase in distance of the markings caused by the air pressure at d_f_.

The opening width of the actuator was determined visually using a camera and two black tape markings attached at each end of the actuator, as is shown in Figure 4A. The pressure supply into the air bladder causes the actuator to open, with higher pressures leading to larger opening widths, as shown in Figure 4B. Overhead views of the actuator at different air bladder pressures were taken, in which the pressure was increased from 0 to 150 Kpa in steps of 10 kPa. The image processing software ImageJ (National Institute of Health, United States) was used to determine the opening width from the photographs taken of the actuator. The profile of the opening width from the taken photographs of the actuator. The profile of the opening width of the actuator in response to different operating pressures of its fabric air bladder component can then be elucidated.

In addition to the opening width, a finite element (FE) model was developed to gain quantitative insights into the forces involved. This is because in the opening width characterization, the loading conditions of the actuator during inflammation of the air bladder are complex, and the force of opening of the actuator consists of the embedded endoskeleton ring. The FE model only considers a half-ring structure representing the endoskeleton, with applied external loading representing the forces exerted by the air bladder. Only half of the endoskeleton ring is modeled by setting symmetric boundary conditions on the middle cross-section because the mechanical conditions are symmetrical to the midline.

**FIGURE 5:**
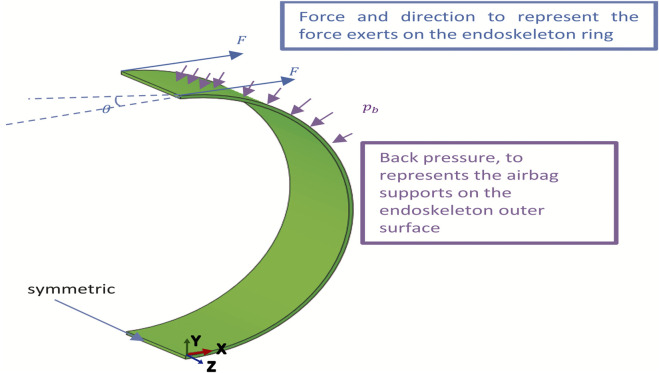
Numerical model for simulating the mechanical behavior of the endoskeleton ring upon pressurization during the opening width characterization experiment. External load and boundary conditions of the endoskeleton strip embedded in the self-securing actuator are defined.

Figure 5, where *F* represents the pneumatic force on the end of the endoskeleton ring and *pb* represents the pneumatic pressure on the external surface of the endoskeleton. Material parameters were obtained directly from a simple tension test conducted on the PVC material of the endoskeleton, revealing Young’s modulus of 0.89 GPa. The forces’ direction (as defined by θ) is approximately based on the observation in the opening width experiment. A normalized force (F*) is defined as shown in Eq. 1, where F_max_ is the maximum force applied on the ring, and the value of F* was increased linearly from 0 to 1.

F*=FFMax (1)

### 2.6 Development of a self-securing hand exoskeleton

The actuator was applied as a self-securing glove interface within a soft robotic hand exoskeleton. Currently, targeted users of rehabilitative hand exoskeletons have various hand impairments, such as stroke, and the main difficulty is getting their hands inserted within the exoskeleton for it to work. The main idea behind the developed self-securing glove is that the user can don the hand exoskeleton by simply placing their hands onto the glove in its uncurled ‘released’ state and then securing their hands by activating the ‘secured’ and curled state. This can help reduce setup time and caretaker assistance needed for donning and doffing hand exoskeleton for rehabilitation.

Two new actuator types were developed for the self-securing glove, adapted from the basic design of the securing actuator for securing the fingers and the palm. They are termed the finger-securing mechanism and palm-securing mechanism, respectively. Subsequently, the self-securing glove is combined with assistive bidirectional finger actuators (developed in previous work (Low et al., 2017)) to yield the self-securing hand exoskeleton.

#### 2.6.1 Finger-securing mechanism fabrication

**FIGURE 6:**
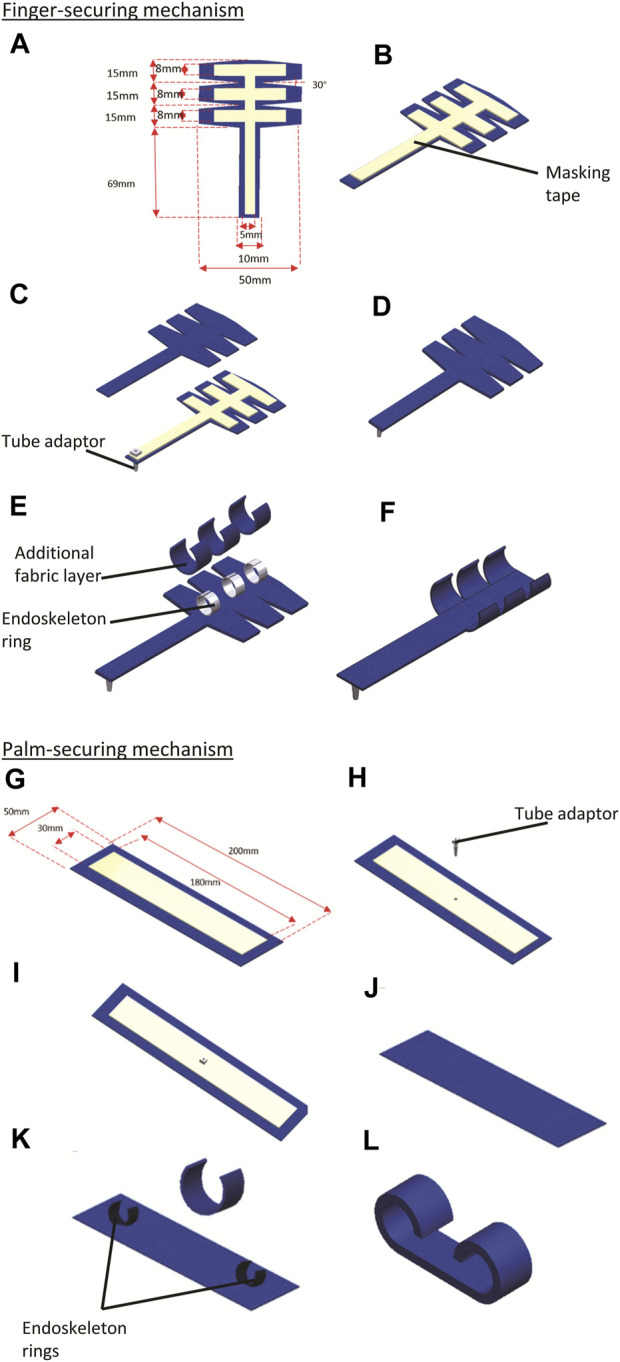
**(A**–**F)** shows the fabrication of the finger-securing mechanism; **(G–L)** shows the fabrication of the palm-securing mechanism. **(A)** Fabric is cut out, and the air channel is dimensioned within the TPU-coated polyester fabric and masked with a masking tape. **(B)** An air inlet was punctured, and the tube adaptor was introduced into the air inlet to allow access to the air channel. **(C)** A s fabric layer with a TPU-coated side facing the masked side of the former layer is placed together and **(D)** heat-sealed to form the air bladder **(E)** Plastic endoskeleton is joined atop of the air bladder via hot glue adhesive, and additional fabric layers were heat sealed to conceal the plastic endoskeleton method. **(F)** The completed finger-securing mechanism in its activated uncurled state. **(G)** The air channel is dimensioned within the TPU-coated polyester fabric and masked with a masking tape. **(H)** An air inlet was punctured. **(I)** Tube adaptor was introduced into the air inlet to allow access to the air channel. **(J)** TPU-coated sides of the fabric are placed atop with another TPU-coated fabric and heat sealed via heat press, getting an air bladder. **(K)** The PVC endoskeleton is joined atop the air bladder at both sides of the bladder via an adhesive method. An additional fabric layer was placed atop the composite, sandwiching the PVC endoskeleton embedded in the air bladder and heat sealed. **(L)** The completed palm-securing mechanism in its inactivated curled state.

The finger-securing mechanism comprises three combined actuators to hold each finger’s phalanges. The air bladder component’s design was adapted and extended to supply pressure to all three actuators-components. Instead of a single rectangular piece, a three-branched pattern was patterned and cut using the TPU-coated polyester fabric. Each brand contains a single securing actuator for the respective phalanx (Figure 6F). Masking tape was lined along the cut fabric sheet’s center line and lengthwise across the three actuators (Figure 6A). A pneumatic tube adaptor was inserted at the end of one of the sheets as an inlet for the air channel (Figure 6B). Another fabric sheet of the same dimension and design was cut out with a TPU-coated side facing the masked side of the former fabric sheet (Figure 6C) and subsequently heat-pressed to form the fabric bladder (Figure 6D). After this, three PVC rings created the three actuator components. The endoskeleton strips were placed on the exoskeleton strips, enveloping them between the fabric strips and the fabric bladder, and heat-pressed to give the completed finger securing mechanism (Figure 6F).

#### 2.6.2 Palm-securing mechanism fabrication

The palm-securing mechanism is larger than the finger-securing mechanism, designed to secure the sides of the palm. The fabrication of this component is identical to that of the single securing actuator except that two endoskeleton rings, similar in dimension to that used in the finger-securing mechanism, were used, one for each side of the palm. Two layers of rectangular-shaped TPU-coated polyester fabric sheet were cut out (Figure 6G). The air channel is dimensioned with a piece of TPU-coated polyester fabric and masked with masking tape. Afterward, an air inlet was punctured in the middle of the masked fabric layer (Figure 6H), and a tube adaptor was inserted into the air inlet to allow access to the air channel (Figure 6I). The TPU-coated sides of both fabric layers were then placed against each other and heat-pressed to form an air bladder (Figure 6J). Subsequently, the PVC endoskeleton C-shape ring was placed atop the fabric air bladder at both sides of the bladder and joined using hot glue adhesive (Figure 6K). The third layer of fabric was subsequently bonded atop the composite using a heat press to reinforce further the endoskeleton strips bonded to the fabric air bladder, forming the palm-securing mechanism (Figure 6L).

#### 2.6.3 Self-securing hand exoskeleton assembly

**FIGURE 7:**
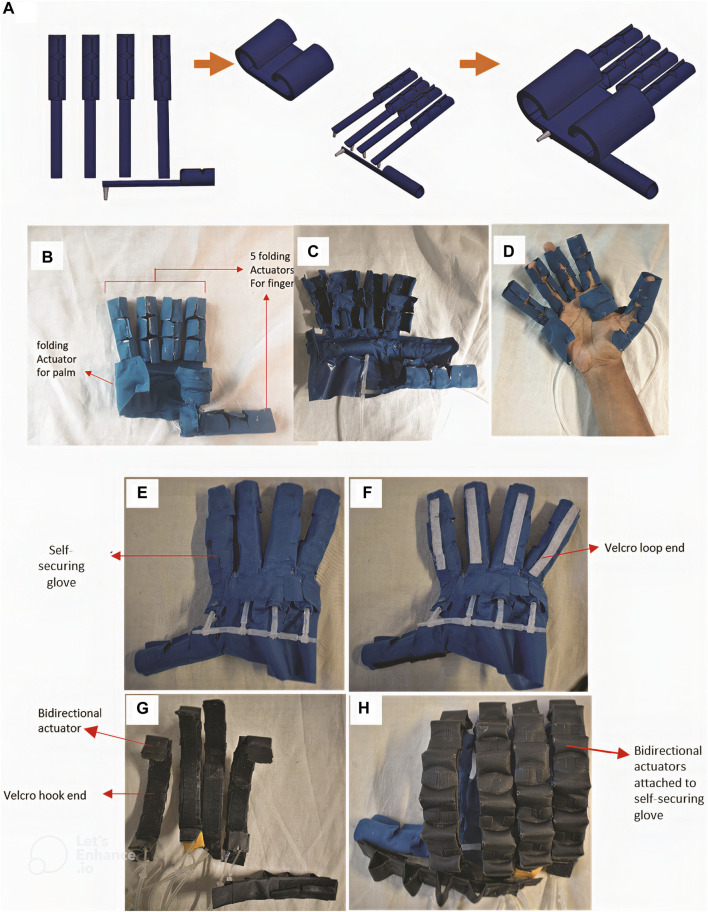
Assembly of the self-securing glove and integration with bidirectional actuators using hook-and-loop straps to give the self-securing hand exoskeleton. **(A)** Fabrication process flow of the self-securing glove; **(B)** the assembled self-securing glove in the depressurized state; **(C)** when in the pressurized opened state during wearing, **(D)** when worn and in a deflated state; **(E)** self-securing glove; **(F)** attachment of loop end finger-securing actuators of the self-securing glove. **(G)** attachment of hook end to bidirectional actuators. **(H)** attaching the bidirectional actuators to the self-securing glove.

First, five finger-securing mechanisms were assembled in the directions of the fingers of a hand, and the palm-securing mechanism was placed at the sides of the palm (Figure 7A). These separate components were then bonded together using a layer of TPU-coated fabric using a heat-press method, giving the full self-securing glove (Figures 7A, B). Upon air pressurization of the self-securing glove, the fingers and palm securing mechanisms uncurl and open, as shown in Figure 7C. This allows users to don the device by aligning their hands on the opened actuator components. Subsequently, the actuator components are depressurized, closing around, and tightly envelopes the hand of the user, as shown in Figure 7D. To doff the glove mechanism, the finger and palm securing mechanisms were inflated, allowing the finger and palm securing mechanisms to uncurl and open. This allows the user to remove the hand from the opened glove mechanism. The next step involves integrating the self-securing glove with bidirectional finger actuators to develop the complete self-securing hand exoskeleton. ‘Hook-and-loop’ strips were used to attach the bidirectional actuators to the self-securing glove (Figure 7E). Five ‘loop’ strips were pasted over the dorsal side of the finger-securing mechanism of the self-securing glove along the axes of each digit (Figure 7F). The ‘hook’ strips were pasted on the five bidirectional finger actuators, as shown in Figure 7G. Then, ‘loop’ strips of the self-securing glove were joined with the ‘hook’ strips of the finger actuators to yield the self-wearing hand exoskeleton, as shown in Figure 7H).

### 2.7 Donning of the self-securing hand exoskeleton

**FIGURE 8:**
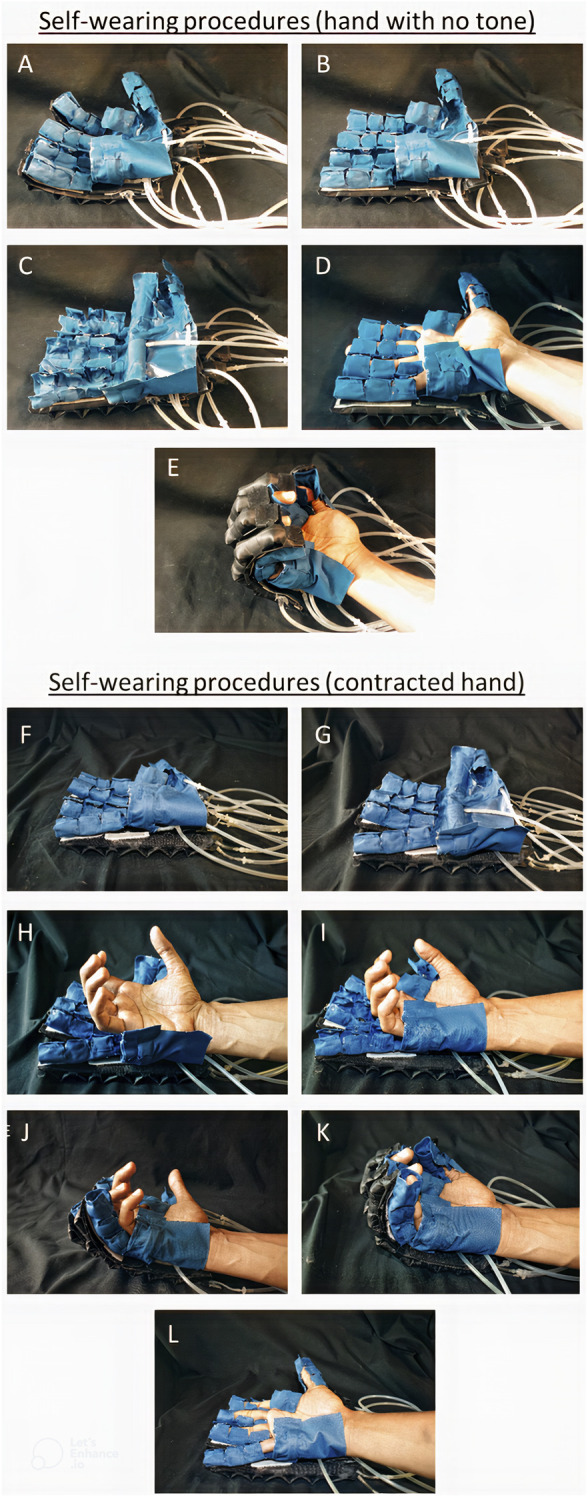
Two workflows of donning the self-securing hand exoskeleton: **(A)**–**(E)** shows the method employed for impaired hand with little muscle tone, whereas **(F)**–**(L)** shows that for impaired hand with high muscle tone due to contraction, or stroke. **(A)**: When the self-securing hand exoskeleton is at default state **(B)** When extension actuators are inflated, the self-securing glove straightens **(C)** the glove is inflated, causing the finger and palm folding actuators to open **(D)** when the glove is deflated with the user wearing it, causing the finger and palm folding actuators to be in a locked state. **(E)** When the extension actuator is deflated, and the flexion actuator is inflated. **(F)** Self-securing hand exoskeleton at its default deflated state. **(G)** self-securing glove mechanism is inflated, **(H)** the clenched hand is placed atop the opened palm securing mechanism. **(I)** palm securing mechanism is then deflated and curled around the palm of the clenched hand, securing the palm. **(J)** Using the slow-increasing pressurization of the flexion component of the bidirectional actuators of the hand exoskeleton, the finger-securing mechanisms are aligned to the clenched fingers of the user. **(K)** Upon alignment, the finger actuators are deflated and uncurled around the finger digits, securing the fingers as well **(L)** Flexion components of the bidirectional actuators are slowly depressurized. In contrast, extension components are pressurized, leading to extension of the fingers of the user’s hand.

For hand impairment without muscular tone, to don the self-securing hand exoskeleton (Figure 8A), the extension component of bidirectional actuators is first pressurized (Figure 8B). This straightens the finger-securing mechanism of the exoskeleton. Next, each actuator component for the phalanges and palm was pressurized simultaneously, opening the self-securing glove mechanism (Figure 8C). Following that, the user aligns their hand over the glove, and the self-securing glove is depressurized, causing them to close and envelope the hand (Figure 8D). The activation of the flexion component of the bidirectional actuator assists in flexing the fingers of the hand, donning the self-securing hand exoskeleton, as shown in Figure 8E. Likewise, the extension component of the bidirectional actuator extends the fingers of the hand, donning the hand exoskeleton.

An alternative method of donning was devised for hand impairment with high muscle tone (Figure 8F–L). First, the extension component of the bidirectional actuators was pressurized, which straightened the finger-securing mechanism (Figure 8F). Next, the self-securing glove was fully pressurized, opening the finger and palm securing mechanisms (Figure 8G). The partially flexed hand of the subject was rested upon the opened glove (Figure 8H), and then the palm securing mechanism was depressurized to curl around and secure the palm. At the same time, the finger-securing mechanisms were still uncurled and opened (Figure 8I). Subsequently, the extension component of the bidirectional actuators was depressurized as the flexion component of the actuators was pressurized. This aligns and flexes the finger-securing mechanisms towards the subject’s flexed fingers (Figure 8J). The finger actuator components were then depressurized to secure the fingers (Figure 8K). Subsequently, the extension component of the bidirectional actuators was pressurized while the flexion component was depressurized, as shown in Figure 8L, showing the donning of the exoskeleton.

### 2.8 Range of motion comparisons

To investigate if the addition of the self-securing glove within the hand exoskeleton would affect its mechanical behavior and outcome of rehabilitation activities performed with it for patients, a visual analysis was carried out to compare the self-securing hand exoskeleton with a previously developed manual, hand-worn soft robotic hand exoskeleton. In particular, the flexed position of the user’s hand within the exoskeleton on activation of the assistive flexion component of the bidirectional actuators was compared when either hand exoskeletons were used.

**FIGURE 9:**
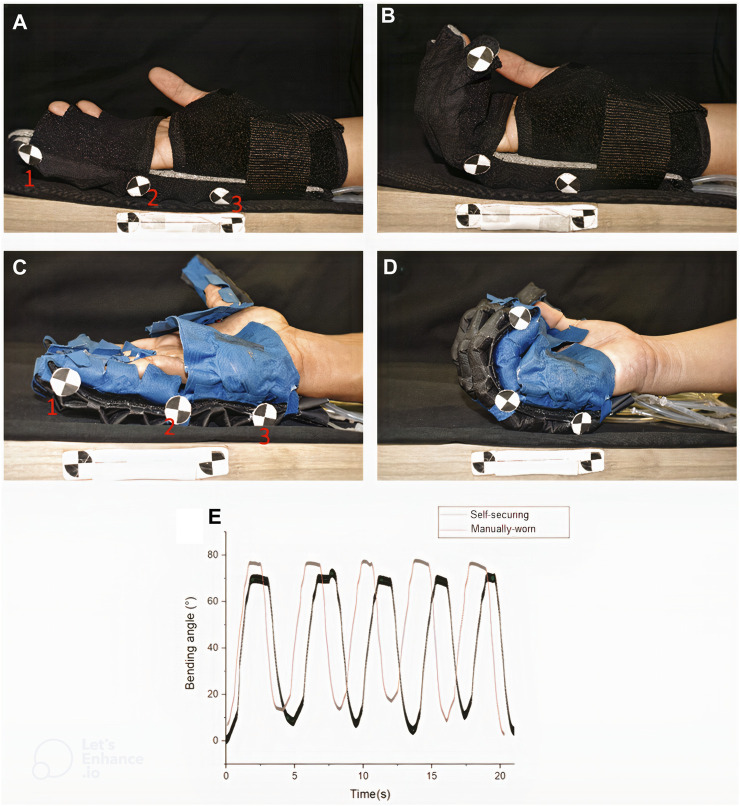
Bending angle comparison between **(A, B)** sleeve-like glove and **(C, D)** self-securing glove **(A)** when extension actuators are fully inflated in both gloves **(A–C)** and when flexion actuators are fully inflated in both gloves **(B)** and **(D–E)** Graphs of bending angle against the time of fingers of self-securing and manual-wearing hand exoskeleton.

Figure 9A shows three checkered trackers (labeled 1,2 and 3) were placed along the little finger of the user, donned with the manual hand exoskeleton and with the self-securing hand exoskeleton (Figure 9C). These trackers allow for the determination of flexion angles by computing the angles enclosed between the line defined by markers 1 and 2 and the line defined by markers 2 and 3. The flexion actuators of both exoskeletons were then alternatively pressurized. They depressurized to perform five cycles of bending and extension of the four fingers for the 20 s (excluding the thumb), causing the fingers to flex and relax (Figure 9E). The movements were video-recorded, and the flexion angles were computed using ImageJ (National Institute of Health, United States).

### 2.9 Gripping test

**FIGURE 10:**
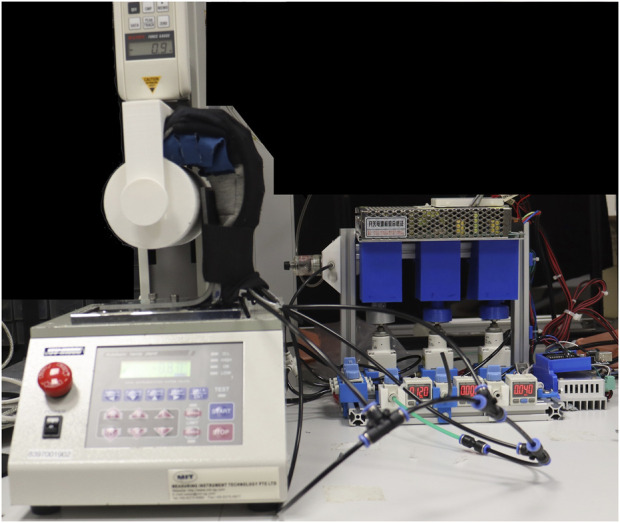
Set-up for the gripping test.

A gripping test was made to measure the gripping force of the assistive glove. The grip force applied by the assistive glove was measured with a universal testing machine (Handy Tester JSV H10000) to obtain normal and frictional grip forces. The assistive glove was pressurized to 120 Kpa to bend and grasp a cylinder that is 80 mm in diameter in a vertical orientation for frictional grip force (Figure 10). The cylinder was then pulled upwards by the universal testing machine at a fixed velocity of 8 mm/s to the point where the assistive glove released the cylinder. The tests were repeated six times, and the results were averaged.

## 3 Results

### 3.1 Locking force characterization test

**FIGURE 11:**
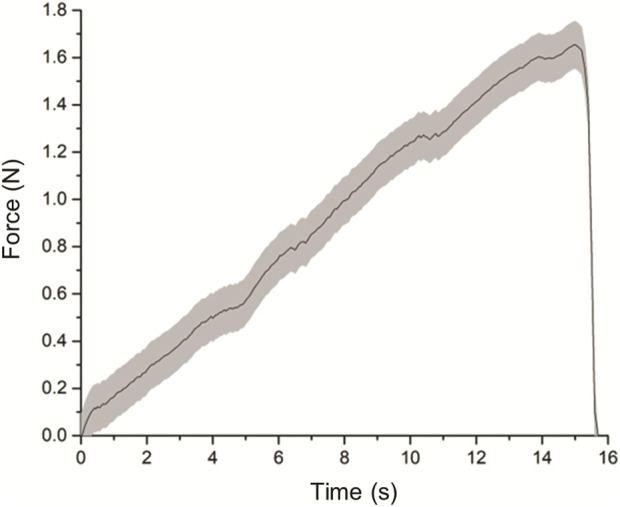
Graph of force exerted on load cell’s claw over time.

Ten measurements for the force exerted on the load cell’s claw were collected for the locking force characterization test. The average result with the standard deviations is presented in Figure 11. The results show that force climbs to a maximum value of 1.63 N (±0.10 N) at the 15-s marl, indicating the claw is dislodging from the actuator before decreasing to 0. This maximum value of 1.64 N (±0.10 N) represents the average locking force of the actuator in the ‘secured’ state.

### 3.2 Opening width characterization test

**FIGURE 12:**
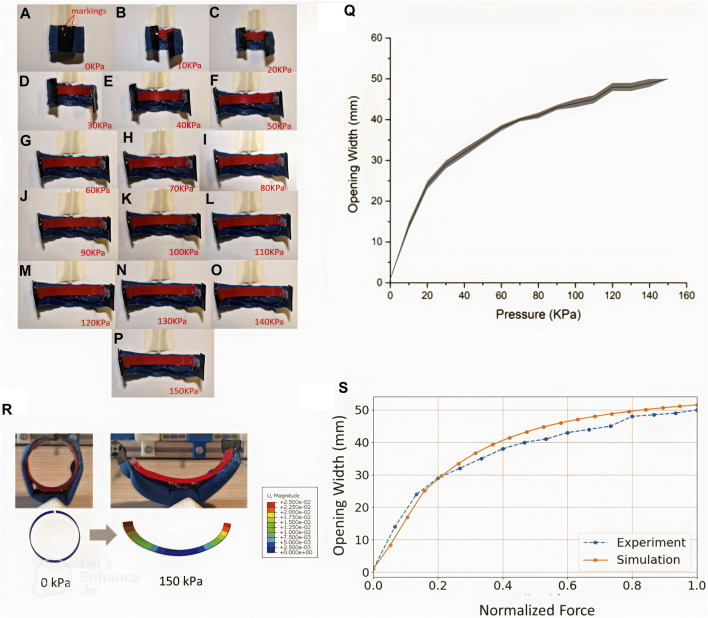
8 Images of the actuator in a pressurized state from 0 kPa to 150 kPa are shown from **(A–Q)** shows the relationship of opening width (mm) against pressure within the actuator (kPa). **(R)** shows a qualitative comparison between the experiment and simulation before and after actuation. **(S)** Comparison of the opening distance between experiment and simulation at different loading levels.

The air bladder’s pressure was increased in a stepwise manner, in steps of 10 k Pa, from 0 KPa to 150 kPa. Figure 12A-P shows the overhead images taken as the actuator was pressurized at the corresponding pneumatic pressures. From these images, the opening width profile in response to the input pressure values was built (Figure 12Q). It was found that after 150 kPa, an opening width of 48 mm was observed from the uncurled state of the pressurized actuator. Any further increase in pressure after 150Kpa leads to an insignificant increase in opening width.

The averaged opening width profile, as shown in Figure 12Q, was used to compare against results from the FE model simulation. Figure 8R shows the initial and final state of the simulation results, which shows a good qualitative match to the experimental images showing the actuator at the minimum and maximum pressures of 0 and 150 kPa, respectively. Figure 12S compares the opening width profile from the characterization test with the FE modeling simulation in which the defined normalized force was increased, and a suitable match was observed. This simulation reveals how the endoskeleton ring behaves under external loading from an inflated air bladder.

### 3.3 Range-of-motion comparisons

The results for the flexion angles during the cyclical bidirectional actuator activations are shown in Figure 9. The bending angle of the self-securing hand exoskeleton was similar to that of the manual-wearing hand exoskeleton, as shown in Figure 9E. These results showed that the addition of the securing mechanism did not compromise the mechanical behavior of the hand exoskeleton.

### 3.4 Grip test

**FIGURE 13:**
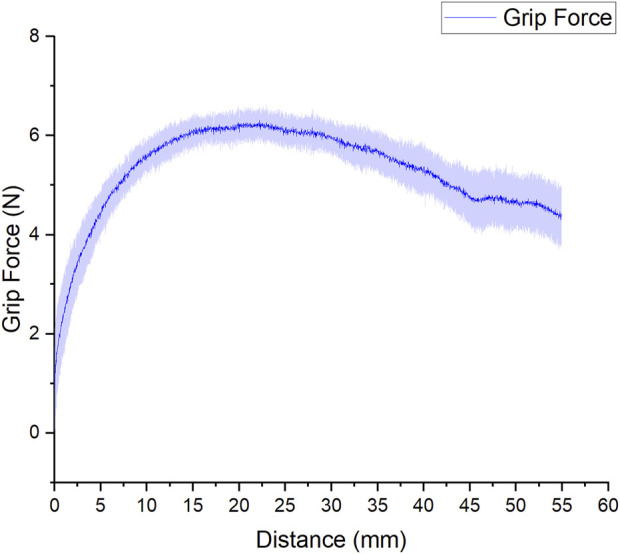
Results of the gripping test.

Several authors used grip tests to measure the gripping force of their respective works (Galloway et al., 2015; Yap et al., 2017b; Correia et al., 2020). The gripping force is an important parameter for assistive hand devices, as during daily activities is common to perform diverse gripping tasks. From the test performed, our results showed a maximal normal grip force of 6 N (±0.35 N) as it can be seen in Figure 13. The normal grip force provided by the self-wearable glove should be sufficient to lift most objects of daily living, which typically do not weigh more than 1 Kg (In et al., 2015; Kang et al., 2016; Popov et al., 2016; Yi et al., 2016; Ullah et al., 2019).

## 4 Conclusion

In this paper, the concept for an endoskeleton-supported hybrid securing actuator was developed to secure wearable devices to the human body. It acts as an interface between the wearable component, such as an actuator or sensor, and the human body segment. The core mechanism involves the mechanical behavior and interaction of two key components-a flexible endoskeleton strip and a pneumatic air bladder. The endoskeleton strip gives shape and defines the configuration of the actuator. At the same time, the pneumatic bladder provides the external forces necessary to bring the actuator from one configuration to the other. As a proof of concept, we have further applied the securing actuator as a self-securing glove within a soft robotic hand exoskeleton to make it more wearable for personnel with impaired hands or hands with a high muscular tone, such as in the case of stroke.

Characterization of the securing actuator was conducted in terms of two quantities—the locking force of the actuator when it is in the default ‘secured’ and curled state; and the opening width, defined as the distance the actuator uncurls upon varying levels of pressurization. From our results, it was observed that the highest force required to overcome the forces was 1.6N, which was considerable in securing small items to the human body, such as the phalanges of the hand. As for the opening width, our experiments revealed that the maximal opening width obtained from the porotype is 48mm, which is sufficient for using it on fingers. Notably, to gain more insights into the forces involved in the opening width experiment, we designed a finite element model and defined the forces of opening the actuator. Our results show a good match between the experiment and model, demonstrating that such modeling techniques may be utilized to optimize actuator design in future studies.

The actuator was further developed into a self-wearing glove for a soft robotic hand exoskeleton. Using the basic actuator, different types of securing mechanisms were developed for the fingers and the palm. The fully assembled self-wearing glove mechanism quickly secures or releases the user’s hand from the hand exoskeleton. In this test, the evolution of bending angle during flexion-extension cycles for the self-securing hand exoskeleton was similar to that of the manually-worn exoskeleton (68 vs 70, respectively). This showed that in addition to force transmission of the finger actuators, the kinematics of the subject’s hand under actuation were also unaffected after integrating the self-securing glove.

The current work was limited to developing self-securing actuators fitting the fingers and the hand sizes. A plausible extension to this work will be to scale the securing actuators to different sizes and extend its application to include apparel, such as wearing a surgical gown, a practical application with which the concept of self-securing can very much help. Also, fatigue testing of the securing actuator and the self-securing glove mechanism would be considered for future work as this is important in determining the life cycle and durability of the actuator in operation.

## Data Availability

The original contributions presented in the study are included in the article/Supplementary Material, further inquiries can be directed to the corresponding author.
